# EyeVolve, a modular PYTHON based model for simulating developmental eye type diversification

**DOI:** 10.3389/fcell.2022.964746

**Published:** 2022-08-26

**Authors:** Ryan Lavin, Shubham Rathore, Brian Bauer, Joe Disalvo, Nick Mosley, Evan Shearer, Zachary Elia, Tiffany A. Cook, Elke K. Buschbeck

**Affiliations:** ^1^ Electrical Engineering and Computer Science, University of Cincinnati, Cincinnati, OH, United States; ^2^ Biological Sciences, University of Cincinnati, Cincinnati, OH, United States; ^3^ Center of Molecular Medicine and Genomics, Wayne State University School of Medicine, Detroit, MI, United States

**Keywords:** eye development, compound eyes, visual system, eye diversity, conserved gene networks

## Abstract

Vision is among the oldest and arguably most important sensory modalities for animals to interact with their external environment. Although many different eye types exist within the animal kingdom, mounting evidence indicates that the genetic networks required for visual system formation and function are relatively well conserved between species. This raises the question as to how common developmental programs are modified in functionally different eye types. Here, we approached this issue through EyeVolve, an open-source PYTHON-based model that recapitulates eye development based on developmental principles originally identified in *Drosophila melanogaster*. Proof-of-principle experiments showed that this program’s animated timeline successfully simulates early eye tissue expansion, neurogenesis, and pigment cell formation, sequentially transitioning from a disorganized pool of progenitor cells to a highly organized lattice of photoreceptor clusters wrapped with support cells. Further, tweaking just five parameters (precursor pool size, founder cell distance and placement from edge, photoreceptor subtype number, and cell death decisions) predicted a multitude of visual system layouts, reminiscent of the varied eye types found in larval and adult arthropods. This suggests that there are universal underlying mechanisms that can explain much of the existing arthropod eye diversity. Thus, EyeVolve sheds light on common principles of eye development and provides a new computational system for generating specific testable predictions about how development gives rise to diverse visual systems from a commonly specified neuroepithelial ground plan.

## 1 Introduction

The fossil record shows that the first organisms with a defined visual system date back ∼500 mya. Since then, a rich diversity of eye types has evolved (Koenig and Gross, 2020) and adapted to a variety of different environments (Land and Nilsson, 2012; Cronin et al., 2014; Meece et al., 2021). Among the simplest prototype “eyes” is a small organ consisting of one photosensitive neuron and an associated pigmented support cell that can detect the presence or absence of light and some directionality (Land and Fernald, 1992; Arendt, 2003; Nilsson, 2009). The more complex eyes that have since evolved are commonly divided into two subtypes: single-chamber (simple) camera-type eyes, like those of vertebrates, and compound eyes, like those of flying insects. The latter are composed of ommatidia, which are relatively simple visual units that typically comprise approximately eight photoreceptors (PRs) that work together to sample one point in space (Paulus, 1989; Warrant and McIntyre, 1993). However, nature’s experiments with arthropods show a range of eye types, from simple to compound, with ommatidia-like units that can be closely clustered or widely distributed. Examples include the ocelli of insects, a set of three simple eyes that may help control flight in some flying insects (Goodman, 1981), and the adult eyes of twisted-wing insects, which combine some of the features of both eye subtypes (Buschbeck et al., 1999).

Developmental and genetic evidence suggests deep conservation in both simple and compound eyes (Gehring, 2001; Arendt, 2003; Oakley, 2003; Kumar, 2010; Koenig and Gross, 2020). This is particularly clear in arthropods, in which many of the simple eyes are known to have evolved from a compound eye ancestor (Buschbeck and Friedrich, 2008; Morehouse et al., 2017), raising the possibility that a unified ancestral developmental framework could underlie this interesting diversity of eye types. Compound eye development is best understood in the model system *Drosophila melanogaster* (Charlton-Perkins and Cook, 2010; Tsachaki and Sprecher, 2012; Pichaud, 2014). This eye is composed of ∼800 precisely organized eye units, called ommatidia (Ready et al., 1976), with a centralized core of eight light-sensitive PR neurons wrapped by four Semper cells (also called cone cells), which are multifunctional cells that secrete the lens mid-pupation and serve glial support roles in adults (Charlton-Perkins et al., 2021). Two primary pigment cells and six secondary and tertiary pigment cells encircle this arrangement, forming a pigment epithelial layer that prevents light scattering between ommatidia. Developmentally, the ocular field is specified during embryogenesis, forming a proliferative pseudostratified epithelial sac (the eye antennal disc) that expands by proliferation through the first two larval stages. During the last (third) larval stage, cells at the posterior tip of the eye disc exit the cell cycle at a perpendicular stripe called the morphogenetic furrow (MF) (Wolff and Ready, 1991b) that moves anteriorly across the eye field, leaving individual clusters of ommatidia in its wake. Cluster formation is a highly organized and stereotyped recruitment process that starts with the emergence of a row of equally spaced ommatidial founder cells (the R8 PRs) followed by sequential recruitment of the remaining PRs (R1–R7) and Semper cells (Tomlinson and Ready, 1987; Kumar, 2012). The MF reaches the anterior portion of the eye during early pupation, finalizing central ommatidial cell recruitment. The final stage of eye patterning involves pigment cell recruitment and the apoptosis of any remaining undifferentiated cells (Cagan and Ready, 1989b).

In arthropods, eye development data outside of *D. melanogaster* are relatively sparse. However, common design principles have been noted in multiple insect species that possess differently structured ommatidia (Friedrich, 2003; Wernet et al., 2015). For example, in beetles, despite lacking an obvious eye imaginal disc like that found in *D. melanogaster*, adult compound eyes develop similarly. This includes an early proliferating primordial epithelium and sequential cell specification of PR cells followed by support cells (Friedrich, 2003; Buschbeck and Friedrich, 2008). Beetles are holometabolous insects, with distinct eye types in larvae and adults. Interestingly, the same general developmental pattern applies to both their adult and larval eyes (stemmata), as exemplified in the sunburst diving beetle (Thermonectus marmoratus) (Stecher et al., 2016). This is particularly remarkable because instead of relatively simple ommatidia, these animals have evolved a set of six dispersed image-forming eyes, with two forward-facing eyes that are particularly large and elaborate, and four smaller and simpler eyes that sample the surrounding visual field (Stowasser et al., 2010; Stowasser and Buschbeck, 2014).

At the genetic level, the very early stages of eye specification involve a deeply conserved transcriptional network (Gehring, 2001; Kumar, 2001; Hsiung and Moses, 2002; Mishra and Sprecher, 2020) comprising the Pax6 “master regulator of eye development” and its downstream retinal determination gene network (RDGN). The latter is contributed to by sine oculis (so), eyes absent (eya), and dachshund (dac), which were originally identified during *D. melanogaster* eye mutant screening (Bonini et al., 1993; Cheyette et al., 1994; Serikaku and O’Tousa, 1994). Evidence for the deep conservation of this network in arthropod eye development (Callaerts et al., 2006) comes from studies on beetles (Yang et al., 2009a; 2009b) and spiders (Morehouse et al., 2017) as well as insect ocelli (Friedrich, 2006). Given the early separation of insects, beetles, and spiders within the arthropod tree of life, these findings are a particularly good indicator of the same deeply conserved eye gene network being capable of generating diverse eye types. Interestingly, many genes in this network share commonalities with vertebrate orthologs (Quiring et al., 1994; Kumar, 2001). Moreover, similarities exist in the patterns of development that specify cell types, and neural circuitry has been suggested to be homologous between vertebrates and invertebrates (Sanes and Zipursky, 2010; Joly et al., 2016), with many relevant transcription factors being deeply conserved (Charlton-Perkins and Cook, 2010; Quan et al., 2012).

**FIGURE 1:**
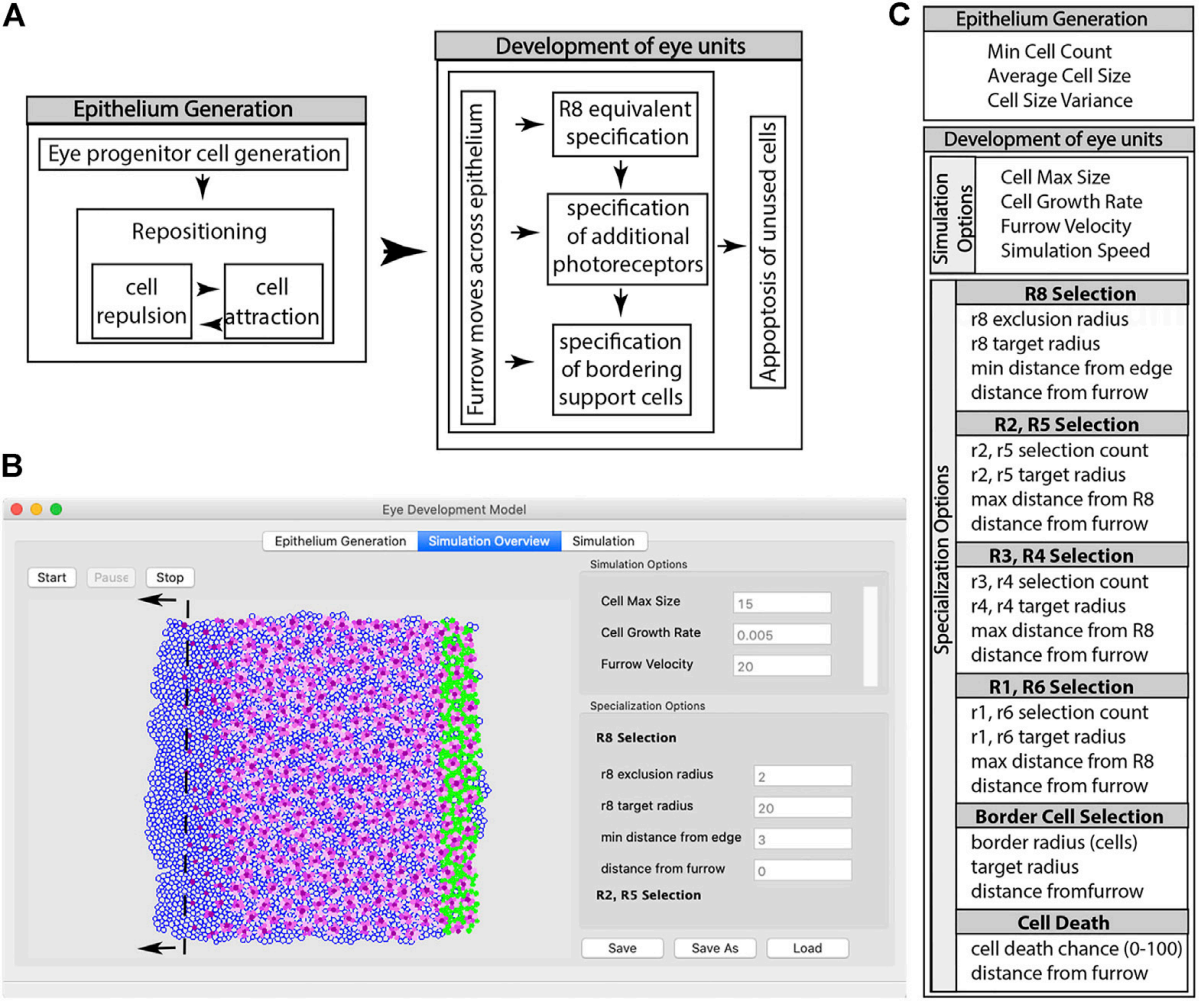
Summary of the steps involved in a simulation using EyeVolve. **(A)**. The first set of steps involves the generation of the precursor epithelium. In the simulation, a user-set pool of eye progenitor cells is generated and repositioned to form a tissue consisting of a single layer of cells. Once the epithelium is generated, the eye unit simulation proceeds, which is initiated by the specification of the R8 founder cell, followed by additional photoreceptor and border cells. **(B)**. As illustrated by a snapshot of the program interface, the model assumes a virtual gradient (dashed line) that moves from right to left and sets the timing for the specification of R8-like cells, additional photoreceptors, and ultimately border cells. The simulation interface is divided into the Epithelium Generation window (left tab), which allows the user to enter parameters for epithelium generation, the Simulation Overview window (middle tab), which allows the user to enter parameters for the development of eye units, and the Simulation window (right tab), which allows the simulation to be viewed. **(C)**. List of modifiable input parameters.

Thus, the question arises of how such deeply conserved developmental gene networks can generate the amazing diversity in eye organization observed among arthropods (Land and Nilsson, 2012). To explore this issue, we developed EyeVolve (Figure 1), a freely available modular PYTHON-based model that uses general developmental concepts from the *D. melanogaster* eye to simulate a 2D model of eye type generation that spans the proliferation and cell recruitment events encompassing early embryogenesis to early pupation. Based on input parameters that influence the size and layout of the precursor epithelium, the spacing of retina-initiating R8-type cells, the number of subsequently recruited PR and support/border/pigment cells, and cell death, the model results in different layouts of eye units, from compound eyes with many units to single-chamber eyes. The model illustrates how the general developmental plan known from *D. melanogaster* may be a universal plan that can lead to the manifestation of diverse eye types and layouts by simply adjusting a few key parameters. EyeVolve was intentionally written to serve as a framework that can be relatively easily expanded to incorporate more specific regulatory steps involved in eye development. In addition, EyeVolve can be used to generate and test hypotheses about how evolution leveraged a deeply conserved developmental plan to give rise to diverse animal eyes.

## 2 Methods

### 2.1 Program structure

EyeVolve is programmed in PYTHON (Python Software Foundation), and is freely available through GitHub: https://github.com/buschbeck-lab/EyeDevelopmentModel/tree/1.0.1.

The program is divided into four modules: the main user interface (‘eye_development_gui’), the simulation interface (“display_2d”), the main simulation (“epithelium_backend”), and a module that contains static parameters (e.g., cell color and line thickness) for relatively easy programming access (‘quick_change’). The simulation mirrors two main events associated with eye development (Hsiung and Moses, 2002): progenitor expansion (epithelia generation) and cell type recruitment (eye unit formation) (Figure 1A). The program first generates an epithelium that consists of cells with a positional coordinate, a radius, and an initial specification as an undifferentiated ocular neuroepithelial cell. In the initial setup, the starting number of epithelial cells is specified using the input parameter “Min Cell Count”, and cells are positioned in close proximity to each other based on repulsion and attraction rules. The Simulation Overview window (Figure 1B) illustrates the cells as they acquire their identity, with the dark purple cells representing the R8-type founder cells, pink cells representing recruited PRs (with earlier recruited cells being darker than later recruited cells), and green cells representing border cells. The MF for the model is an imaginary line that dictates the timing of key events according to simple distance rules that the user sets as parameters through the eye_development_gui. These “furrow events” act on vertical slices of the epithelium at a user-defined distance behind the furrow. Each event marks an important developmental milestone (e.g., the differentiation of a precursor cell into a specific cell type) and is coded as a separate function; thus, as more regulatory processes are identified from animal studies, they can be readily added as future modules. Furrow events, along with cell events and rules for displaying cells, are defined in the quick_change module.

### 2.2 Rationale and biological basis for input parameters

Input parameters are divided into those necessary for generating the epithelium and those that influence the simulation of cell specification (Figure 1C).

#### 2.2.1 Epithelium generation

Following the observation that early proliferating primordial epithelia are common among different eye types (Friedrich, 2003; Buschbeck and Friedrich, 2008), the first module of the program is devoted to the generation of a precursor epithelium, the size of which is defined by the “Min Cell Count” input parameter. Because the simulation of a large epithelium is relatively computation-intensive, the program allows the user to save (and reload) the same epithelia. This also allows the influence of other parameters to be tested on the same starting tissue environment. In addition to cell number, a cell size parameter, “Average Cell Size”, allows the user to adjust the relative size of the precursor cells and the later differentiating cells. Finally, “Cell Size Variance” allows cells to be slightly differently sized. This is important because several of the simulation steps are based on identifying cells by distance to simulate the presence of gradients known to play important roles in the onset of cell differentiation (Gordon et al., 2020). Small differences in cell size break symmetries and, hence, ambiguities. Indeed, some variation in cell size exists in nature, which appears to be regulated by not only genetics (Huang et al., 1999; Weinkove, 1999) but also external factors such as temperature (Azevedo et al., 2002).

#### 2.2.2 General simulation options

After the initial setup of the starting epithelium, the user is moved to the Simulation Overview tab to input parameters that influence how the undifferentiated cells expand by proliferation and become specified. The first parameter, “Cell Max Size”, defines the maximal size to which each cell can grow. The second parameter, “Cell Growth Rate”, controls the rate and timing at which the cells grow in each simulation cycle. Larger numbers result in cell division within undifferentiated epithelial cells. The next input parameter, “Furrow Velocity”, controls how far the virtual line representing the MF advances across the epithelium from right to left during each simulation cycle, mimicking the posterior to anterior movement of the MF of *D. melanogaster* (Wolff and Ready, 1991b). Finally, the user can set the “Simulation Speed”, represented as cycles per second, which is mostly important for slowing down simulations of small tissues to observe the progression of differentiation more readily.

#### 2.2.3 Eye unit founding photoreceptors cell selection

In *D. melanogaster*, the specification of individual eye units within the compound eye begins with the selection and spacing of the initial founder cell, PR R8 (Tomlinson and Ready, 1987; Kumar, 2012). EyeVolve allows the user to define the minimal spacing between founder cells (‘r8 exclusion radius’), the size to which this cell type can grow (“r8 target radius”), and how far away the cells can be seeded from the edges (“min distance from edge”). The last parameter ensures that a sufficient number of unspecified cells remain around the founder cell to allow the formation of full units, a feature, that is, particularly important for larger eye units. Finally, “distance from furrow”, defines the distance from the MF at which a founder cell differentiates into an R8 PR. The process is visualized by a change from an empty progenitor to a purple R8 cell.

#### 2.2.4 Additional photoreceptors specification

In *D. melanogaster*, the major light-sensing PRs, R1–R6, are sequentially recruited as R2/R5, R3/R4, and R1/R6 cell pairs (Wolff and Ready, 1991b). As a default, EyeVolve color codes these functionally related cell types with different shades of pink and purple, with later recruited cells becoming gradually paler in color. The parameters for each of these cell types include how many are recruited (“rX selection count”), how large they grow (“rX target radius”), how far they are allowed to form from the founder PR (“max distance from R8”), and when they are formed relative to the MF (“distance from furrow”). Note that the last retinal cell types to be specified in *D. melanogaster*, the R7 PR and four Semper cells, are not yet included in EyeVolve, as their natural positions above the PRs cannot be adequately visualized in this 2D rendition.

#### 2.2.5 Border cell selection

In *D. melanogaster*, border cells are represented by pigment cells that are recruited during early pupation from the remaining pool of epithelial progenitors to separate individual eye units. In EyeVolve, this border affects how well units are separated and can be influenced by changing the “border radius”, which determines how many layers of cells are recruited, the “target radius”, which defines the maximum size to which these cells grow, and the “distance from furrow”, which sets the timing for differentiation.

#### 2.2.6 Cell death

In *D. melanogaster*, eye unit borders are refined by eliminating any remaining unspecified cells through programmed cell death (apoptosis) (Wolff and Ready, 1991a; Miller and Cagan, 1998; Rusconi et al., 2000; Monserrate and Brachmann, 2007). In EyeVolve, the timing and speed of cell death are set using the input parameters “distance from furrow” and “death chance”, which is the probability that each cell will die. If ‘death chance’ is set to 0, cells do not die off; instead, if the remaining cells are allowed to continue to divide, the spacing between eye units increases.

EyeVolve is based on the developmental logic that underlies *D. melanogaster* compound eye formation. Therefore, the default settings of the program will generate that type of compound eye.

### 2.3 Optimizing parameters for genus-specific eye layouts

To illustrate the similarities between real and simulated *D. melanogaster* eye development, the eye antennal discs of late third instar larvae from a common lab stock, yw67, were stained with DAPI (at 1 µg/ml to visualize nuclei) and phalloidin (at 1:400 to visualize actin-rich structures). The discs were then mounted in Vectashield (Vector Laboratories) and imaged with a Zeiss LSM 700 confocal microscope. The obtained images were processed using Adobe Photoshop 2022.

To test the applicability of EyeVolve to other eye organizations, we empirically modified input parameters to generate eye organizations that mimic those found in arthropods, using scanning electron micrographs for comparison. The specific parameters used in these simulations are summarized in Table 1.

**TABLE 1:** Input parameters used by EyeVolve to produce the layouts illustrated in Figures 2–4.

	Drosop hila	Strepsi ptera	Lepido ptera larvae	Diving beetles	Ocelli	One eye - expansion	One eye fitsion
Min cell count	10,000	7,000	1,000	7,000	10,000	5,000	5,000
Average cell size	8	10	10	10	10	10	10
cell size variance	0.1	2	2	2	2	2	2
Cell max size	15	15	15	15	25	15	15
Cell Growth rate	0.005	0.005	0.005	0.005	0.01	0.005	0.005
Furrow Velocity	20	20	20	20	10	20	20
simulation speed	100	100	100	100	10	100	100
r8 exclusion radius	2	10	10	25	28	40	0.8/1
r8 target radius	20	10	10	10	20	10	10
min distance from edge	3	10	4	13	25	30	8
distance from furrow	0	0	0	0	0	0	0
r2,r5 selection count	2	35	2	50	150	800	2
r2,r5 target radius	20	20	20	20	30	20	20
max distance from R8	2	50	2	50	200	600	2
distance from furrow	100	100	100	200	100	400	100
r3 r4 selection count	2	35	2	100	150	800	2
r3, r4 target radius	20	20	25	18	30	25	25
max distance from R8	2	50	2	50	200	500	2
distance from furrow	150	200	200	400	150	800	200
r1,r6 selection count	2	35	2	50	150	800	2
r 1,r6 target radius	25	20	25	20	30	25	25
max distance from R8	4	50	4	80	200	600	4
distance from furrow	200	300	300	600	200	1,000	300
border radius	1	2	1	2	5	1	1
target radius	20	20	20	20	15	20	20
distance from furrow	1,000	400	400	800	550	2000	400
death chance	30	20	20	20	10	20	20
distance from furrow	1,200	1,000	1,000	1,000	600	2,200	1,000

## 3 Results

### 3.1 Recreating the eye layout of *D. melanogaster*

**FIGURE 2:**
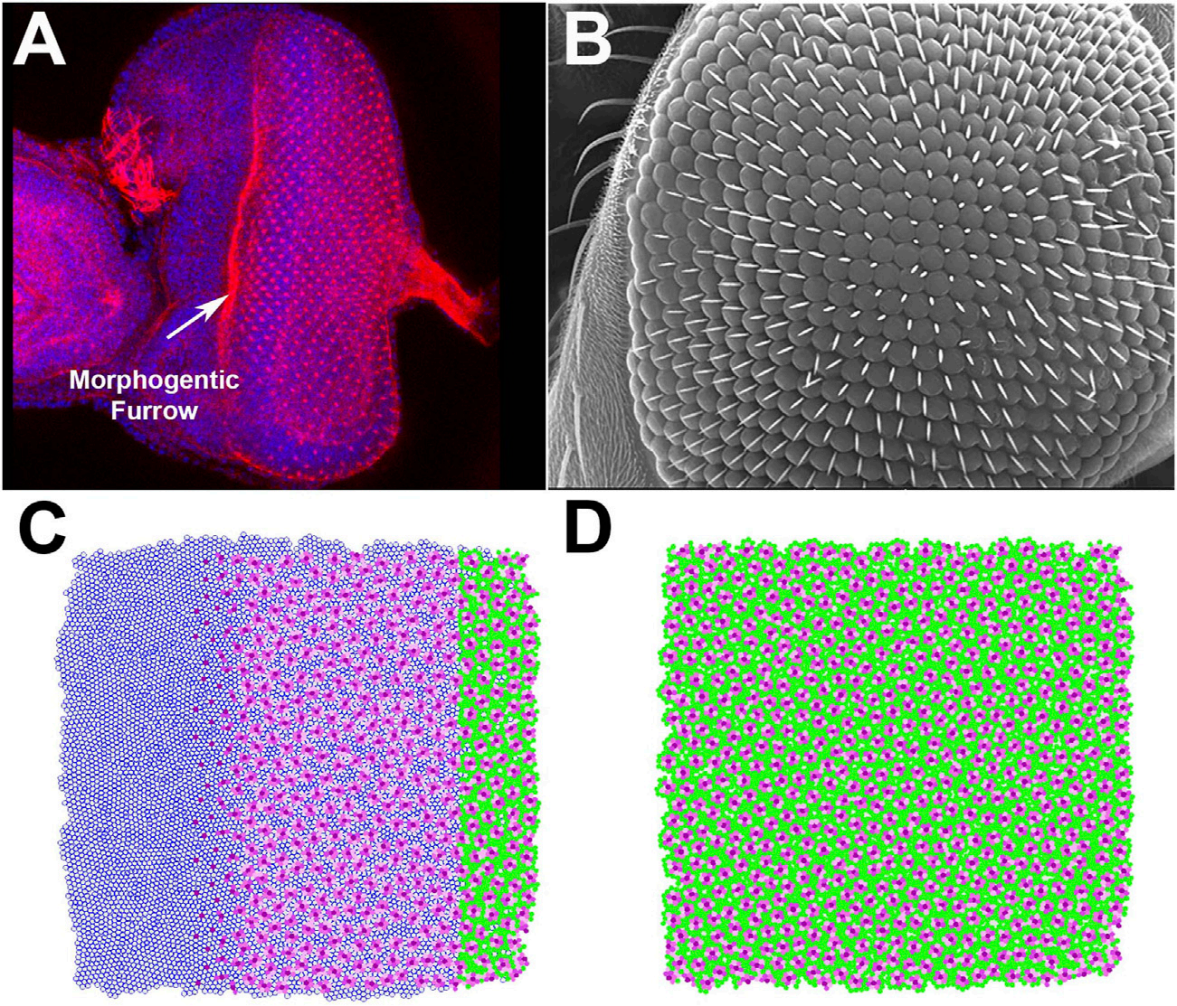
Proof-of-principle test of the ability of EyeVolve to recapitulate the eye layout of *Drosophila melanogaster*. **(A)**. DAPI and phalloidin staining of the developing adult eye at the late third larval instar stage. **(B)**. Scanning electron micrograph of the surface of an adult *Drosophila* eye, illustrating the precise and regular positioning of ommatidia. **(C)**. EyeVolve image taken during the simulation to illustrate how ommatidia develop progressively from right to left. **(D)**. EyeVolve image of a fully developed compound eye.

In *D. melanogaster*, eye unit (ommatidia) formation starts in mid-stage third instar larvae with the formation of the MF at the posterior edge of the precursor epithelium, which then progresses anteriorly, one ommatidial row at a time (Wolff and Ready, 1991b). Following the cell cycle arrest initiated by the MF, R8 founder cells establish ommatidial spacing and initiate the stereotyped recruitment of PRs (R1–R6) as R2/R5, R3/R4, and R1/R6 pairs. This is followed by the recruitment of the R7 PR, four Semper cells, and border cells, which also include bristle cells. Any excess cells are then removed, allowing for a relatively regular layout of ommatidial units. To visualize this process, we stained a developing eye disc with DAPI (to picture the distribution and position of cells) and phalloidin (to stain actin-rich PRs) (Figure 2A). The fully developed *D. melanogaster* eye is characterized by a highly regular array of ommatidia (Figure 2B).

To model *D. melanogaster* in EyeVolve (see methods for details on the computational logic and input parameters), we started with a 10,000 cell epithelium and set the “r8 exclusion radius” to 2. As the program finds the next set of R8 cells on the basis of their minimal distance to existing ones, the ommatidial units develop relatively close to each other in a roughly hexagonal pattern. The default simulation (Figure 2C and Supplementary Movie “*Drosophila* example”) illustrates the emergence of ommatidial units from a precursor epithelium (Figure 2A). Although the output of our model does not achieve the same level of precision in regard to the regularity of the ommatidial units, it does achieve a good approximation of the typical hexagonal arrangement (Johnson, 2021). Additional factors (that are not currently implemented in the program) are known to play important roles in achieving the level of precision manifested in *D. melanogaster*, including proper adhesion between cells (Bao et al., 2010), precise levels of cell size (Kim et al., 2016), and appropriate elimination of excess cells (Wolff and Ready, 1991a; Miller and Cagan, 1998; Rusconi et al., 2000; Monserrate and Brachmann, 2007). However, less-regular compound eyes have been observed for other insects, such as in the Madagascar hissing cockroach (Gromphadorhina portentosa) (Mishra and Meyer-Rochow, 2008) or in the ventral portion of certain male butterflies (Uchiyama et al., 2013), suggesting that imperfect organization from the default parameters may be relevant to some (including more ancestral) compound eyes.

### 3.2 Reproducing diverse eye layouts

**FIGURE 3:**
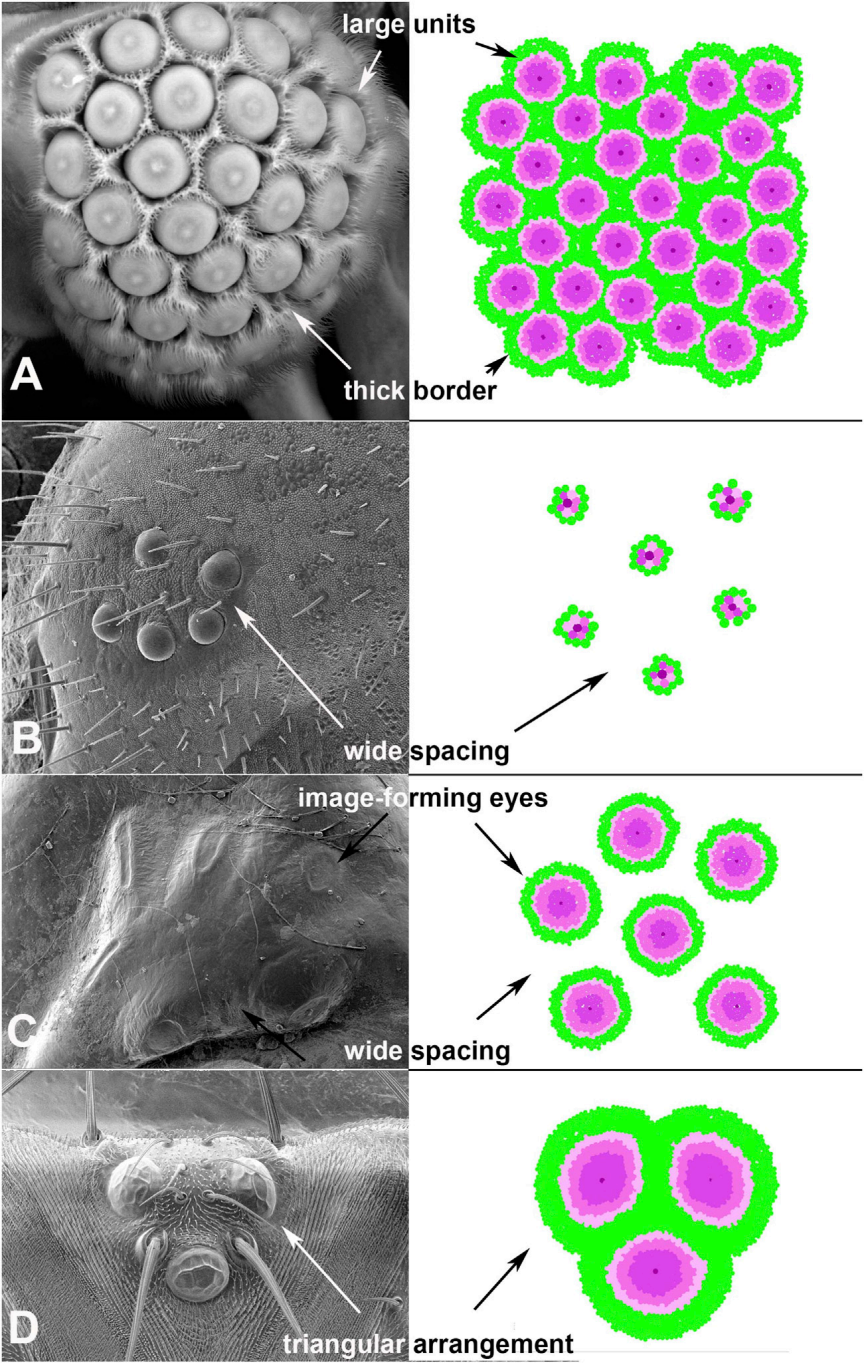
Adjustments to input parameters allow EyeVolve to develop authentic eye layouts. **(A)**. Male twisted-wing insects have “chunk vision”, an organization of relatively large units, each with a small image-forming eye, that is, wrapped with bristles. **(B)**. Lepidoptera larvae tend to have six ommatidial-like units that are spread far apart, as exemplified by a fifth instar of Papilio (Image credit: Kentaro Arikawa). **(C)**. Dytiscus larval eyes are characterized by a cluster comprising six relatively small image-forming eyes. **(D)**. The dorsal ocelli of insects, exemplified here in *Drosophila melanogaster*, typically comprise three units in a triangular arrangement. This reconstruction is oriented with development having progressed in the dorsal-to-ventral direction.

Given the deeply conserved patterns in eye development, the use of this system is expected to successfully develop a wide range of eye types. To test whether EyeVolve could reproduce other eye layouts, we examined a variety of insects with different eye arrangements and adjusted the input parameters to optimally reproduce them (Table 1). The first example is based on Xenos peckii (Strepsiptera), a twisted-wing insect characterized by a combination eye in which relatively large image-forming units are bordered by a field of bristles. Each eye unit samples a small image of a portion of the visual field, with the complete image being assembled in a compound-eye-like array (Buschbeck et al., 2003). Based on the phylogenetic position of X. peckii among insects that have compound eyes and the finding that these eyes follow a similar posterior-to-anterior developmental succession, it has been proposed that the eyes of X. peckii evolved from an ancestral compound eye (Buschbeck, 2005). Therefore, they represent a good model for the transition between ommatidial-like units and image-forming units. The most important adjustments for this eye type were more distantly spaced founder PR cells (by changing the “r8 exclusion radius” from 2 to 10, in order to seed eye units further apart from each other) and an increase of secondarily recruited PRs and border cells. Specifically, for each type of secondarily recruited PR cell selection counts were increased from 2 to 35. Intrinsic to the model, the increased size of each unit also lead to the recruitment of more border cells and our model was able to produce a layout similar to that found in nature (Figure 3A).

Next, we turned our attention to larval eyes, a hallmark yet highly varied feature of holometabolous insects. Larval eyes, known as stemmata, are a particularly interesting group of eye types, as they are derived from compound eyes but have diverse manifestations, ranging from compound eyes to sophisticated image-forming eyes (Buschbeck, 2014). In most orders, larvae have five to seven stemmata with molecular developmental similarities to compound eyes (Friedrich, 2006, 2008). Even within Lepidoptera (moths, butterflies), there is considerable variation in the placement and organization of stemmata (Singletonsmith and Philogène, 1981; Lin et al., 2002). As a test case, we considered the stemmata of the butterfly Papilio xuthus. This family of animals develop larval eyes that are characterized by six relatively simple units, each following the general organization of ommatidia (Paulus, 1989)) and spaced relatively far apart (Figure 3B). For EyeVolve to recapitulate this general pattern the most important adjustment was a relatively small (1,000 cell) epithelium (to reflect the overall smaller size of the eye field) with the same “r8 exclusion radius” that was used for Strepsiptera, and the same secondary photoreceptor recruitment values that were used for *Drosophila*.

The second set of stemmata simulated by EyeVolve (based on a 7,000 cell epithelium) is reminiscent of the larval eyes of the diving beetle Dytiscus (Figure 3C and Supplementary Movie “6 medium sized eyes example”). Here, each of the six stemmata consists of a relatively small, image-forming eye (Günther, 1911), and the units are somewhat separated from each other, as in the case of Papilio. The most major adjustment for a simulation was a large “r8 exclusion radius” (50) and a large number of secondarily recruited photoreceptors. Again, our model was able to reconstruct the general layout relatively well, except for the unusual oval shape exhibited by some of the Dytiscus eye units.

Finally, as many insects are characterized by three median ocelli, we also used EyeVolve to simulate the development of such median eyes as exemplified for the ocelli of *Drosophila* (Figure 3D). These relatively large units have many PRs and prominent lenses. With a precursor epithelium of 10,000 cells, the recruitment of 450 R1-R6 type PRs (for an extended retina), and the recruitment of extra border cells for better separation (by setting “border radius” to 5), the model successfully developed a triad of units that upon completing cell growth were organized in a tight triangular arrangement, reminiscent of the ocelli of many insects (Goodman, 1981).

### 3.3 Formation of camera-type eyes through expansion or fusion

One interesting finding is that EyeVolve can form individual camera-type eyes through either the expansion of individual units by adding PRs (as already demonstrated for ocelli) or the fusion of multiple units. Evidence exists that both of these mechanisms have occurred in the evolution of arthropod eye diversity (Buschbeck and Friedrich, 2008). For example, based on a developmental study on the large, elongated image-forming larval eyes of the water beetle T. marmoratus, it is likely that the evolution of this complex eye type involved the addition of PRs, expanding from an ancestral ommatidial-like unit (Stecher et al., 2016). On the other hand, there is also evidence of unit fusion, as exemplified by the stemmata of the flour beetle *Tribolium castaneum* (Liu and Friedrich, 2004). In addition, fusion likely played a role in the development of the telescopic lens system within the compound eyes of Mysid shrimp (Nilsson and Modlin, 1994).

**FIGURE 4:**
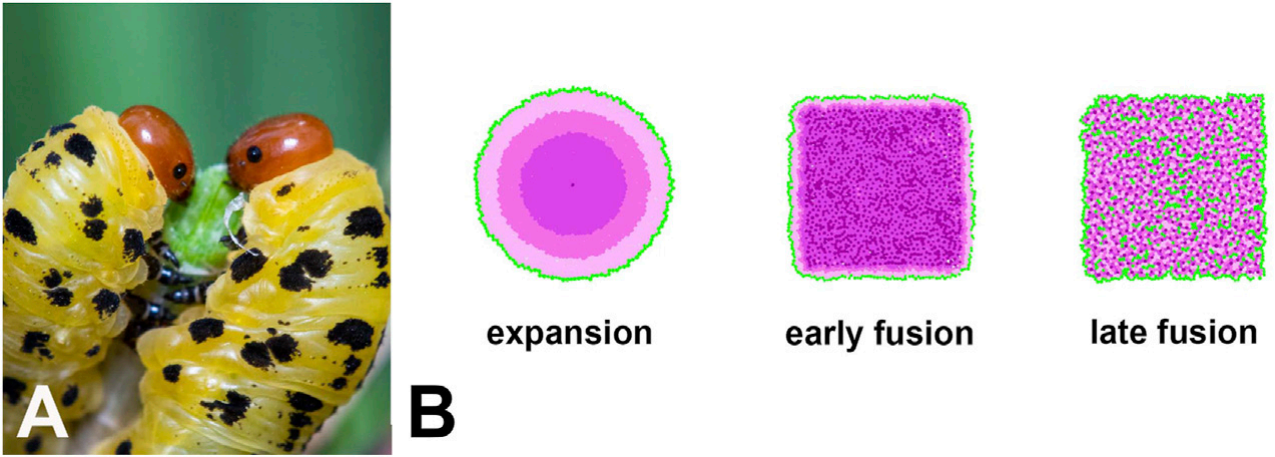
**(A)**. One example of a single-lens eye that likely evolved from an ancestral compound eye is that of sawfly larvae (Image credit: Ryan Ridenbaugh). **(B)**. Simulations of different ways in which an extended retina can form within the framework of our model as an illustration of how single-lens eyes could arise. In the expansion example, development starts with a single R8-like receptor that then recruits several layers of additional receptors, thus mimicking the evolution of a single-lens eye from an ommatidial-like precursor. This mode of development leads to the concentric placement of different photoreceptor types. The second and third examples are based on fusion at different stages of development. If fusion occurs early in development, support cells and late developing PRs are restricted to the periphery, resulting in a single-lens eye with mixed photoreceptor types. If fusion occurs later in development, all PRs and some support cells are also integrated into the retinal mosaic. Note that the square shape of the two fusion examples is related to the current limitations of our model in regard to the shape of the precursor epithelium rather than actual limitations in eye shape.

Depending on the input parameters (see Table 1), EyeVolve generated camera-type eyes through both mechanisms, yet led to dramatically different distributions of PR subtypes (Figure 4B). For example, if the eye forms from a single R8 founder and expands through the recruitment of additional PRs, then the different subtypes of PRs are recruited in concentric circles. On the other hand, when development starts with relatively closely spaced founder PRs, with each recruiting a typical set of PRs that fuse into one retina (depleting epithelial cells prior to the formation of unit borders), the PR array tends to consist of a mosaic of different receptor types. In addition, if founders are seeded very closely to each other (early fusion), late-recruited PR types can be pushed to the periphery (see Supplementary Movie “One large eye early fusion example”). In contrast, if founders are seeded slightly further apart (late fusion), then all the PR types and even some support cells are integrated into a retinal mosaic. In both cases, the eye is delimited by support cells. As shown in Figure 4A, sawfly (Hymenoptera) larvae are characterized by a large bilateral single stemma. Although eye development in this group has not yet been studied, based on the PR layout (Meyer-Rochow, 1974), we postulate that fusion plays an important role.

### 3.4 Diverse eye layouts arising from limited input parameters

**FIGURE 5:**
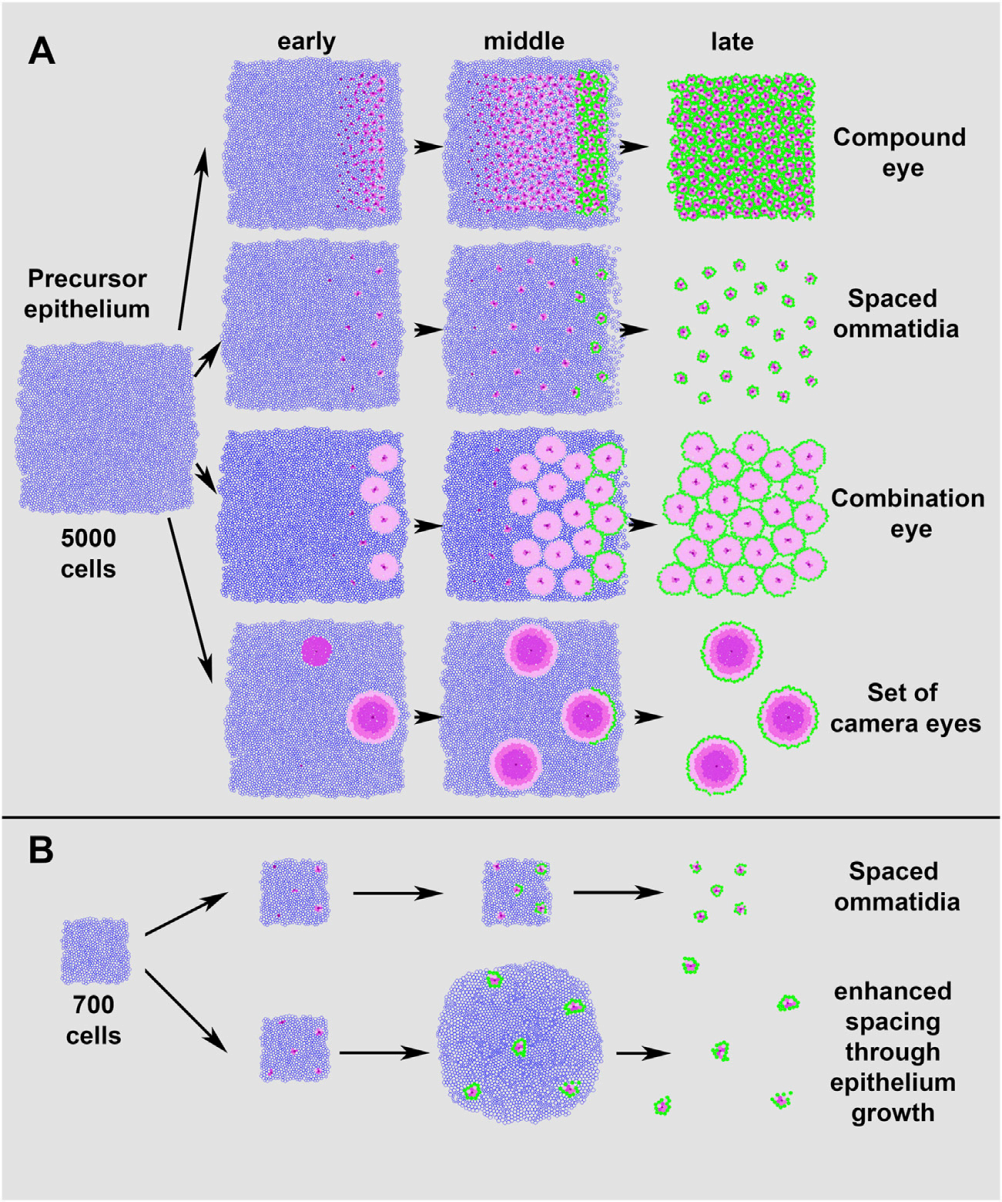
Simulations of different eye layouts from a single precursor epithelium. Adjusting only a few of the input parameters allows EyeVolve to achieve very different eye layouts. **(A)**. Simulation of eye layouts from a 5,000 cell epithelium. The top row represents the typical ommatidial layout of a compound eye, with cell differentiation starting at the posterior edge and progressing anteriorly. In the second row, the ommatidial units are the same but spaced further apart, as achieved by simply increasing the “r8 exclusion radius” from 3 to 10. The third row demonstrates how the addition of photoreceptors (in this case, “r1, r6 selection count” was increased from 2 to 100) leads to the development of an eye that combines the optical features of compound eyes and image-forming lens eyes. The final row shows how a set of camera-type eyes might develop in a similar manner with a few additional changes, such as seeding R8 cells at a larger distance but further from the edge, increasing the number of photoreceptors (R2–R6-like cells), allowing for a larger recruitment distance from R8, and increasing the distance from the furrow for each step. **(B)**. Simulation of eye layouts from a 700 cell epithelium. The top row represents the ommatidial layout obtained by reducing the epithelium starting size and decreasing the “min distance from edge” (from 8 to 3), as compared to the spaced ommatidial layout in **(A)**. The bottom row illustrates that delayed cell death (“distance from furrow” increased from 1,000 to 100,000) results in enhanced spacing after cell specification.

To test which input parameters are essential for different eye layouts, we created a 5,000 cell epithelium and generated various eye layouts by minimally adjusting the input parameters based on our previous observations (Figure 5A). Surprisingly diverse layouts could be recovered by changing only three key parameters (see Supplementary Table S1 for the full list of tested parameters). First, the “r8 exclusion radius”, which determines how far apart founder cells form from each other, defined the degree to which the centers of eye units were separated from each other. Second, the number of recruited PRs established the relative size of each eye unit. Finally, as the eye unit size increased, it became necessary to prevent founder cell seeding at the edges, accomplished using the “min distance from edge” parameter, to ensure that sufficient progenitor cells surrounded the founder to generate a complete eye unit.

Figure 5A illustrates the simulation of a typical compound eye (generated with the following critical settings: “r8 exclusion radius” = 2; each PR subtype = 2; “min distance from edge” = 8). From these settings, increasing the “r8 exclusion radius” 5-fold (from 2 to 10) led to a compound eye organization in which the ommatidia-like units were spaced relatively far apart. A further increase in the PR number (in this case, the “r2, r6 selection count was changed from 2 to 100) resulted in a Strepsiptera-like combination eye with fewer but larger closely situated image-forming units. The model offers the flexibility to select the number for each PR subtype, and we found that similar eye types were formed regardless of the subtype (not illustrated). In addition, EyeVolve could develop a cluster of image-forming eyes by tweaking these two parameters further (“r8 exclusion radius” = 25, “r2, r6 selection count” = 300) and increasing the minimum distance from the edge (from 9 to 15).

Finally, our model includes two additional parameters that further increase eye diversity (Figure 5B). The first is related to the size of the precursor epithelium, with smaller epithelia giving rise to fewer eye units, as illustrated by the “spaced ommatidia” simulations in Figures 5A,B, which differ only in the starting size of the epithelium (5,000 and 700 respectively) and the minimum distance from the edge (8 and 3). Moreover, Figure 5B illustrates that delayed cell death (“distance from furrow” changed 100-fold from 1,000 to 100,000) leads to units drifting apart after the cells are specified. As the units are pushed apart, some of the specified cells are slightly displaced because mechanisms for tight cell adhesion are not yet implemented in EyeVolve.

## 4 Discussion

EyeVolve is an eye development simulation program based on the development of the *D. melanogaster* compound eye, which is the best-known example from genetic studies. As such processes are deeply conserved (Gehring, 2001; Arendt, 2003; Oakley, 2003; Kumar, 2010; Koenig and Gross, 2020), we found that EyeVolve is able to not only simulate *D. melanogaster* eye unit formation but also predict vastly different eye organizations by adjusting only a few input parameters. Previous models have been aimed at capturing relatively specific aspects of eye development, including early spatio-temporal dynamics (Fried et al., 2016) and specific cell fate choices (Graham et al., 2010; Lubensky et al., 2011). In contrast, EyeVolve is geared toward integrating the three major cellular events involved in fly eye development to generate a fully patterned eye and to allow rapid testing that can address which regulatory processes lead to the formation of other eye types. The results reported here indicate that quite diverse eye types present in arthropods can be reproduced by manipulating very few steps in the process. These key steps include precursor epithelium growth, founder PR spacing, PR recruitment, border cell recruitment, and apoptotic pruning of undifferentiated cells (Figure 5). These findings are intriguing in light of the communalities that have already been observed in the RDGN (see introduction), suggesting that the parameters addressed by EyeVolve could be tightly linked to, and downstream to that well-studied network. In many cases, information already exists as to how specific genes and pathways contribute to each of these steps in *D. melanogaster*. Therefore, EyeVolve serves as a tool to illuminate how specific genes and pathways might contribute to the diversification of eye layouts.

### 4.1 Key steps in the development of diverse eyes

This section summarizes the most important steps that we identified as being critical to the generation of diverse visual system layouts. Each step is associated with a developmental process, that is, relatively well understood in *D. melanogaster*, and specific examples are given of how genes are known to relate to these processes. For each step, we also formulate testable predictions that would allow us to evaluate our model in diverse arthropod visual systems.

#### 4.1.1 The size of the precursor epithelium sets the stage

The initial steps of eye development involve the development of a precursor epithelium, which in *D. melanogaster* corresponds to the eye imaginal disc. First established in the embryo, the eye disc grows by proliferation throughout the first two larval instar stages before the onset of retinal differentiation in the early third instar (Casares and McGregor, 2021). In addition, a single additional round of proliferation occurs during PR recruitment (between R3/R4 and R1/R6), a feature that has not yet been implemented in EyeVolve for simplicity. Regardless of when cell division occurs, the size of the eye field sets the stage for ‘how much eye’ can be developed. In *D. melanogaster*, the proliferation rate (and therefore size) of this tissue is under the precisely regulated, region-specific control of well-known molecular cascades, including the growth-promoting Notch signaling (dorso-ventral axis) and JAK/STAT pathways (Baonza and Freeman, 2005; Domínguez and Casares, 2005), and differences in the size and shape of the precursor epithelium have already been recognized as important factors (Lobo-Cabrera et al., 2021). Altering the proliferation rate of progenitors in the precursor epithelium is one option for size regulation, as has been shown in mutant flies with the transmembrane protein Crumbs. These flies have enlarged compound eyes due to overactive Notch signaling during early eye development (Richardson and Pichaud, 2010). Although the genetic control of epithelium size is an active area of study (Casares and McGregor, 2021), environmental factors such as temperature and food availability have also been shown to affect eye size in *D. melanogaster* (Azevedo et al., 2002), and flies with smaller eyes have been demonstrated to have poorer vision, especially in darker environments (Palavalli-Nettimi and Theobald, 2020). As early eye developmental processes related to the precursor epithelium are relatively conserved (see introduction and (Casares and McGregor, 2021)), we expect that epithelium regulation is an important contributor to arthropod eye diversity. An interesting example here is stemmata (Buschbeck, 2014), the larval eyes of holometabolous insects that evolved from compound eyes which exhibit a remarkable diversity in eye size and visual system layout. For the very large and functionally elaborate stemmata of sunburst diving beetles (T. marmoratus) (Stowasser and Buschbeck, 2014), it has been shown that the cluster of six eyes on each side of the head originates from a relatively large, shared precursor epithelium (Stecher et al., 2016). We predict that this equally applies to the stemmata of other Coleoptera or Lepidoptera, as modeled by EyeVolve (Figures 3B,C). More broadly, we predict that the size of the precursor epithelium is generally indicative of the size of the fully developed visual system, irrespective of eye type. Based on our model, we furthermore predict that clusters of eyes with a more distributed arrangement (e.g., the sophisticated camera-type eyes of tiger beetle larvae (Gilbert, 1989; Toh and Mizutani, 1994; Toh and Okamura, 2001, 2007) and possibly the clusters of spider eyes (Morehouse et al., 2017)) develop from a common precursor epithelium.

#### 4.1.2 The placement of founder PR cells (R8 in *D. melanogaster*) is instrumental for the number and spacing of developing eye units

The first PR for each unit is the founder R8, which is central to organizing the remainder of the cells in each eye unit. The relative spacing of the founder PR cells is therefore an important factor in determining the number and spacing of the visual units that can arise from the epithelium. In *D. melanogaster*, and likely more generally in arthropod compound eyes (Friedrich, 2003; Buschbeck and Friedrich, 2008), founder cells differentiate immediately behind the MF (Tomlinson and Ready, 1987) in an evenly spaced configuration (Baonza et al., 2001). R8 recruitment is essential for eye unit development, as it is required to initiate the addition of more PR cells (Frankfort and Mardon, 2002). For this reason, and based on subsequent testing with EyeVolve, we postulate that the number and spacing of founder PR cells is a key determinant for eye layout across evolution.

Due to the absence of ommatidial units in *D. melanogaster* R8 mutants, the genetic circuitry required for R8 selection and differentiation is well established (Frankfort and Mardon, 2002). Moreover, such studies led to the discovery that the same circuitry drives neurogenesis and founder cell specification in all metazoa and that mutations in this network result in vision loss. Selecting evenly spaced founder cells within a pluripotent field of cells requires the integration of multiple signaling and downstream transcription factors. Short-range furrow advancement and cell cycle arrest require Hedgehog and BMP signaling, whereas regional neurogenic potential and centralized proneural fate determination involve Notch-Delta signaling. The downstream transcriptional effectors of the Notch pathway that ultimately define the founder cell are encoded by two related and cooperative factors: Atonal (fly ato, human ATOH7) and Daughterless (fly da, human TCF4) (Jarman et al., 1993). A single point mutation in the DNA-binding domain of ato leads to failed R8 specification and subsequent eye loss, whereas ato overexpression leads to multiple founder cells (Jarman et al., 1994; Frankfort and Mardon, 2002). Additional evidence for the deeply conserved role of this gene comes from experiments in which atonal orthologs of different organisms (mice, lancelets, and annelids) were used to replace the innate gene in *D. melanogaster*, leading to the rescue of R8 specification, albeit at variable rates (Weinberger et al., 2017). EyeVolve allows the simulation of such scenarios, wherein a relatively tight specification of R8 results in a layout similar to that of the *D. melanogaster* compound eye (Figures 2C,D), whereas more distant spacing is necessary for some other eye types (Figure 3). One of the predictions of our model is that the organization of diverse arthropod eyes relies on the presence of an R8-like founder cell in each of the eye units. This could be verified by looking for ato and senseless expression or potentially through knockdown of these genes in different species. In addition, it is expected that ablation of the founder PR would prevent the development of the entire eye unit. The model further predicts that the manipulation of founder cell spacing affects both the number and spacing of eye units that can develop. Given that some stochastic processes are involved in founder cell selection, our model also predicts that minor differences in the number and placement of individual eye units can occur between individuals (and even within an individual), especially when many units develop from a single epithelium. Finally, it is expected that seeding founder cells close to the edge of the precursor tissue would give rise to incomplete eye units, and indeed in some compound eyes, small units are observed at the edge. In *D. melanogaster*, it is also known that partial ommatidia are removed through wingless/Wnt-dependent elimination (Kumar et al., 2015).

#### 4.1.3 The recruitment of additional PRs lays the foundation for unit size

In *D. melanogaster*, PR recruitment involves a complex network of molecular cascade, short-range signaling from Notch and the epidermal growth factor receptor (EGFR) with cell-type-specific transcriptional regulators. Notch, for example, participates in epithelial proliferation and ato-positive cell selection at the MF, whereas the resulting ato-positive R8 cells stimulate EGFR signaling in neighboring progenitors through the short-range activation of Spitz (Spi), a diffusible ligand for EGFR (Baonza et al., 2001). The reiterative use of EGFR and Notch in each subsequent set of recruited cells drives the stepwise differentiation of the remaining PRs with a combinatorial transcription factor code: Rough in R2/R5, Seven-up and Spalt in R3/R4, Seven-up and BarH1 in R1/R6, and Prospero/Runt/Spalt in R7 (Baonza et al., 2001). Thus, it is expected that these factors are under evolutionary pressure and that alterations can relatively easily influence the number of specific PR types that are recruited. Accordingly, EyeVolve allows the user to independently define the number of each of the recruited PR pairs. In arthropods, for example, the highly reduced *D. melanogaster* stemmata (the Bolwig organ) contain only R8-like cells, whereas in other cases, the PR number is greatly increased within a given eye unit (Buschbeck, 2014). For instance, the larval stemmata of T. marmoratus have two large tiers of PR cells that form sequentially (Stecher et al., 2016), as predicted by the EyeVolve expansion simulation (Figure 4B). The PR types within the tiers differ in terms of opsin expression and spectral sensitivity (Maksimovic et al., 2009, 2011). Adult visual organs also have varying PR numbers. A particularly drastic example is the combination eye of X. peckii, in which approximately 100 PRs are present in each unit (Buschbeck et al., 1999). EyeVolve closely recapitulates this unusual eye organization, as well as the larval stemmata of another diving beetle, Dytiscus (chosen for comparison here because their eye units are more uniform in size than those of Thermonectus), and the *D. melanogaster* ocelli (Figures 3A,C,D). As an extreme case, we could also simulate the development of a single camera-type eye, resembling those found in certain stemmata and ocelli. Interestingly, this could be done in two different ways: through a single founder cell, leading to a layered organization of surrounding cells, or through many founder cells that are seeded so close together that there is no space for border cells (see next section). A prediction from our model is that it should be relatively easy to regulate eye unit size and PR distributions within units molecularly, following the outlined patterns. For example, we predict that arthropods with very differently sized eyes develop by simply recruiting variably sized sets of PRs. Finally, we predict a trade-off between the number and size of units that can develop from an epithelium of a given size; when some eyes get bigger, others necessarily have to be smaller or fewer eye units will arise from that epithelium.

#### 4.1.4 Support cells are required to define boundaries and separate eye units

The next step in eye development is the recruitment of support cells. In *D. melanogaster*, once PRs have been specified, they influence the fate of the remaining cells, turning neighbors into support cells (Kumar, 2012), leading to additional support cells in the presence of additional PRs (Cagan and Ready, 1989b; Freeman et al., 1992). Immediately following PR cell specification, Semper cells, which are close relatives of PRs, are recruited from a subset of precursor cells (Kumar, 2012; Charlton-Perkins et al., 2021). Semper cells then recruit primary pigment cells via the combined influence of EGFR and Notch signaling pathways (Nagaraj and Banerjee, 2007), which then recruit interommatidial cells (IOCs) through a tightly regulated process requiring the roughest gene (Araujo et al., 2003). As illustrated by Sparkling (spa) mutant flies, disruption in these differentiation events results in severely miss patterned units, including abnormally formed lenses and fused ommatidia (Fu and Noll, 1997). The fusion of visual units, for example, due to the insufficient recruitment of IOCs, has been hypothesized to explain the evolution of camera-type eyes in some holometabolous insect larvae (Buschbeck, 2014) such as the sawfly (Hymenoptera) (Figure 4A). The cross-section of these larval stemmata is composed of a camera-type eye under which lies a retina, that is, reminiscent of that in a compound eye (Meyer-Rochow, 1974). The involvement of fusion in the evolution of single-chamber eyes likely also applies to the giant single-lens eyes of the mysid shrimp Dioptromysis (Nilsson and Modlin, 1994). As EyeVolve is currently 2D, different support cell types are combined into a single category, simply referred to as border cells. Despite this simplification, the model captures the principle of additional PRs leading to additional support cells and can simulate the development of a camera-type-eye via the fusion of multiple discrete visual units (Figure 4B). This results in a retinal mosaic in which different PR types are interspersed. Based on EyeVolve, we predict that the recruitment of support cells is a key determinant of whether some separate functional units remain or all eye units fuse into one eye, with the loss of border cell recruitment being particularly important for the evolutionary transition between compound eyes and single-chamber eyes. Conversely, extra border cells can contribute to a more distinct separation of units, as observed in Strepsiptera (Figure 3A). As bristles are a subset of border cells, this raises the possibility that cell fate changes within the border cell population can also contribute to eye diversification.

#### 4.1.5 The apoptosis rate of remaining cells molds the final eye layout

In the *D. melanogaster* compound eye, once all necessary cells have differentiated, apoptosis of the remaining cells fine tunes the final ommatidial layout. This process is regulated by a precise interplay between Notch (promoting apoptosis (Cagan and Ready, 1989a; Parks et al., 1995)) and Ras (impeding apoptosis (Miller and Cagan, 1998)) pathways. These phenotypes suggest that the rate of apoptosis is another important parameter that could mediate evolutionary differences in eye unit organization. Indeed, in our model, the cell death rate influences the final position of the developing units. In cases in which the eye units are widely separated (e.g., the larval eyes of Lepidoptera, Figure 3B), it is expected that more of the separated cells will persist. Conceptually, eye units could be pushed even further apart if mitosis occurred, as illustrated in Figure 5B. Further, EyeVolve predicts that it should be impossible to add PRs within the eye field after the eye units are fully formed. Indeed, in hemimetabolous insects, compound eyes grow by adding new units peripherally rather than between units that have already formed (Friedrich, 2008). Even in fossil radiodonts, compound eye units were added at the periphery (Paterson et al., 2020). Finally, consistent with these expectations, it was recently demonstrated that the eyes of tiny juvenile jumping spiders have approximately the same number of PRs as those of their much larger adult counterparts (Goté et al., 2019).

### 4.2 Model applicability

Our simulation is based on the developmental events for *D. melanogaster* compound eyes. Nevertheless, simple tweaks to the input parameters of EyeVolve reproduce a variety of arthropod eye types, further supporting the hypothesis that these different visual systems evolved from a common ground plan. This is particularly apparent for the lateral eyes in insects and likely equally valid for those of crustaceans due to their relatively related ancestry and known patterns of eye development (Harzsch and Hafner, 2006). The model likely also applies to insect ocelli, which are considered important for flight control (Kastberger, 1990). Ocelli typically consist of three camera-type eyes that each contain a relatively large number of PRs (Stark et al., 1989). Ocelli are thought to share developmental plans with insect compound eyes (Brockmann et al., 2011; Zhou et al., 2016), despite having diverged at least 500 mya (Friedrich, 2006). Their primordial epithelium derives from an eya and so-positive region situated antero-dorsally in the eye disc (Blanco et al., 2009) that eventually fuses with the contralateral side to form a fused medial and two independently derived lateral ocelli. Much variation exists in the size and placement of ocelli between different taxa (such as Hymenoptera, Odonata, and Diptera (Ribi and Zeil, 2018) and even between diurnal and nocturnal bees (Warrant et al., 2006). Based on EyeVolve, differences in placement could be related to the number of cells that remain between eye units or the rate of apoptosis in the final steps for ocelli patterning, a topic that warrants further exploration.

Arachnids are particularly interesting because they show impressive diversity in the size and layout of image-forming eye units, which likely have also evolved from a compound eye ancestor (Morehouse et al., 2017). In scorpions, eye units are typically relatively simple, but the clusters exhibit considerable layout diversity (Loria and Prendini, 2014), which could easily be explained by some minor changes in the key parameters discussed above. Interestingly, intra- and interindividual variation also occurs, which is consistent with stochastic processes leading to the placement of founder cells, as implemented in EyeVolve. For these reasons, systematic studies of scorpion eye development could be particularly insightful. Finally, as mentioned in the introduction, arthropod eye development involves deeply conserved genes that also play a role in vertebrate eye development (Quiring et al., 1994; Kumar, 2001; Quan et al., 2012), with the possibility of some homologous aspects (Sanes and Zipursky, 2010; Joly et al., 2016). Noticeably, some of the features predicted by EyeVolve share similarities with the observed features of vertebrate retinas, including central vs. peripheral PR subtype formation (Figure 4B) and stochastically distributed PR subtypes. Thus, it is likely that at least some aspects of EyeVolve could be informative for vertebrate eye developmental studies, although further investigation is required.

### 4.3 Opportunities for model expansion

EyeVolve has allowed us to capture common principles and key parameters that can be altered to give rise to diverse and relatively realistic eye layouts. This program is specifically designed in a modular way, so that individual steps can be relatively easily expanded, for example, by incorporating specific molecular logic as it becomes available. Indeed, some important aspects have already been modeled and could be incorporated into the broader but simpler model presented here. This includes factors that influence the precursor epithelium, such as cell proliferation (Amore and Casares, 2010; Lobo-Cabrera et al., 2021) and transcription factor activity (Fried et al., 2016; Gavish et al., 2016; Jörg et al., 2019). Other processes that have been modeled include MF dynamics, Delta-Notch-dependent lateral inhibition during R8 selection (Formosa-Jordan et al., 2013; Fried et al., 2016) and subsequent cellular patterning (Graham et al., 2010; Larson et al., 2010; Lubensky et al., 2011). Our hope is that EyeVolve will expand to include increasingly realistic biological representations as more detail emerges on how different genes contribute to the processes currently captured as general steps. In addition, although EyeVolve already captures the basis for different eye layouts, additional input parameters could render the shape of the precursor epithelium, facilitate the position-specific emergence of differently sized eye units, and influence patterning through differential cell–cell adhesion properties. Other anticipated improvements include moving from a 2D to a 3D model, which would allow us to define different types of support cells, such as Semper cells, which are positioned above PRs in most compound eyes. We hope that these qualities will make EyeVolve an important tool for further exploring the general rules of eye development and how such rules have shaped the evolution of animal eyes.

## Data Availability

EyeVolve is freely available through GitHub (https://github.com/buschbeck-lab/EyeDevelopmentModel/tree/1.0.1) and a complete list of input parameters that were used for simulations presented in this paper is available in the Supplementary Material.

## Supplementary material

Supplementary Table S1 Input parameters used by EyeVolve to produce the layouts illustrated in Figure 5. Values that differ from those for compound eye construction are indicated in bold.
