# Supplementary material for: EyeVolve, a modular PYTHON based model for simulating developmental eye type diversification
Source: Front Cell Dev Biol. 2022 Aug 26;10:964746. doi: 10.3389/fcell.2022.964746 (PMC9459020; doi:10.3389/fcell.2022.964746)
Supplement: Supplementary file 2 [file Table1.docx]

Supplementary Table1: Input parameters used by EyeVolve to produce the layouts illustrated in Fig. 5. Values that differ from those for compound eye construction are indicated in bold

|  | Compound eye | Spaced ommatidia | combination eye | cluster of camera eyes | few units | few units far apart |
| --- | --- | --- | --- | --- | --- | --- |
| Min cell count | 5000 | 5000 | 5000 | 5000 | **700** | **700** |
| Average cell size | 10 | 10 | 10 | 10 | 10 | 10 |
| cell size variance | 2 | 2 | 2 | 2 | 2 | 2 |
| Cell max size | 15 | 15 | 15 | 15 | 15 | 15 |
| Cell Growth rate | 0.005 | 0.005 | 0.005 | 0.005 | 0.005 | 0.005 |
| Furrow Velocity | 20 | 20 | 20 | 20 | 20 | 20 |
| simulation speed | 100 | 100 | 100 | 100 | 100 | 100 |
| r8 exclusion radius | 2 | **10** | **10** | **25** | **10** | **10** |
| r8 target radius | 10 | 10 | 10 | 10 | 10 | 10 |
| min distance from edge | 8 | 8 | 8 | **15** | **3** | **3** |
| distance from furrow | 0 | 0 | 0 | 0 | 0 | 0 |
| r2,r5 selection count | 2 | 2 | 2 | 2 | 2 | 2 |
| r2,r5 target radius | 20 | 20 | 20 | 20 | 20 | 20 |
| max distance from R8 | 30 | 30 | 30 | 30 | 30 | 30 |
| distance from furrow | 100 | 100 | 100 | 100 | 100 | 100 |
| r3, r4 selection count | 2 | 2 | 2 | 2 | 2 | 2 |
| r3, r4 target radius | 25 | 25 | 25 | 25 | 25 | 25 |
| max distance from R8 | 30 | 30 | 30 | 30 | 30 | 30 |
| distance from furrow | 200 | 200 | 200 | 200 | 200 | 200 |
| r1, r6 selection count | 2 | 2 | **100** | **300** | 2 | 2 |
| r1, r6 target radius | 25 | 25 | 25 | 25 | 25 | 25 |
| max distance from R8 | 30 | 30 | 30 | 30 | 30 | 30 |
| distance from furrow | 300 | 300 | 300 | 300 | 300 | 300 |
| border radius | 1 | 1 | 1 | 1 | 1 | 1 |
| target radius | 20 | 20 | 20 | 20 | 20 | 20 |
| distance from furrow | 800 | 800 | 800 | 800 | 800 | 800 |
| death chance | 20 | 20 | 20 | 20 | 20 | 20 |
| distance from furrow | 1000 | 1000 | 1000 | 1000 | 1000 | **100000** |
